# Integrative taxonomic revision of three *Camellia* species from section *Tuberculata* (*Camellia*, Theaceae) by morphological, anatomical, palynological, and molecular evidence

**DOI:** 10.1186/s40529-025-00489-5

**Published:** 2026-01-19

**Authors:** Weihao Gu, Mingtai An, Chao Yan, Xu Xiao, Zhaohui Ran, Zhi Li

**Affiliations:** College of Forestry, Guizhou University, Guiyang, 550025 China

**Keywords:** Section *Tuberculata*, Complex species, Taxonomic revision, Integrative taxonomy, Species delimitation

## Abstract

**Background:**

The section *Tuberculata* (*Camellia* L.) comprises 18 species, forming a monophyletic group with unique “tuberculate-wrinkled fruit pericarp” morphological characteristics. However, the interspecific relationships within this section remain poorly resolved. A notable taxonomic controversy involves *Camellia lipingensis*, *C. zengii*, and *C. rhytidocarpa*, which were previously considered conspecific. On the basis of extensive population surveys conducted in their type localities, we identified significant morphological disparities among these three taxa. To comprehensively clarify their taxonomic status and relationships, we conducted an integrated study incorporating morphology, micromorphology (leaf epidermis and pollen), and molecular systematics (cpDNA and nrDNA ITS).

**Results:**

Evidence from morphology, anatomy, palynology, and molecular systematics consistently supports the treatment of *Camellia zengii* as a heterotypic synonym of *C. lipingensis*, while confirming the distinct species status of *C. rhytidocarpa*. Morphological analysis revealed continuous variation in key traits: Leaves lanceolate (6.42–12.50 × 2.16–4.45 cm); floral parts with 6–9 rounded sepals, 3–5 hairy styles, and 2.2–4.1 cm long filaments; fruit subglobose (diameter 2.24–3.18 cm), ovary 3-4-loculed (1 seed per locule). Anatomical and pollen characteristics are conservative: The leaf epidermal stomata are elliptical (39.9–41.2 × 31.4–36.7 μm), with a density of 62–86 per mm², and the pollen is nearly spherical (polar axis 36.7–37.8 μm/equatorial axis 40.3–41.3 μm, P/E ratio 0.87–0.91). Molecular phylogenetic analyses confirmed that *Camellia lipingensis* and *Camellia zengii* form a strongly supported monophyletic group (ML/PP = 100/1.00; clade Ⅰ), with *Camellia rhytidocarpa* forming a separate clade sister to it. The chloroplast genomes of the three taxa are conserved in structure, with consistent chloroplast genome structures (157,029, 157,029, 157,048 bp; GC 37.3%; containing 87 protein-coding genes, 37 tRNA genes, and 8 rRNA genes).

**Conclusions:**

This study conclusively resolved the taxonomic delimitation among these closely related species within sect. *Tuberculata.* We propose the synonymization of *Camellia zengii* with *C. lipingensis* and confirm that *C. rhytidocarpa* is a distinct species, a conclusion robustly supported by congruent evidence from multiple disciplines. Our research provides a practical framework for reconstructing phylogenies in morphologically complex plant groups, informs conservation prioritization, and contributes to the development of standardized species delimitation protocols.

**Supplementary Information:**

The online version contains supplementary material available at 10.1186/s40529-025-00489-5.

## Introduction

Section *Tuberculata* of the *Camellia* genus is distinguished from other *Camellia* species by its unique “tuberculate-wrinkled fruit pericarp” (Chang and Ren 1991; Min and Zhong 1993; Chang 1998). In the early 20th century, the renowned botanist, Prof. Chien (1939), discovered a taxon with tuberculate ovaries and pericarps during fieldwork in Jiading district, Sichuan province, China, and named it *Camellia tuberculata* S.S. Chien. Sealy (1958) later placed it in section *Heterogenea* on the basis of a literature review. It was not until 1981 that Hung-Ta Chang established the sect. *Tuberculata*, which is based on the diagnostic “tuberculate-wrinkled fruit pericarp” that initially included 6 species, with 12 additional species reported over the next decade. In 1991, Chang subdivided the section into two subsections (subsect. *Tuberculata* Chang and subsect. *Nudicarpa* Chang) on the basis of ovary pubescence. Tianlu Min (1993), a famous *Camellia* taxonomist, revised this classification process and divided 18 species into 6 species, 6 varieties, and 1 form (Min and Zhong 1993). Notably, the taxonomic treatments proposed by these two researchers were developed under the methodological constraints of their time, relying principally on herbarium specimens with limited field validation and minimal integration of multidisciplinary taxonomic evidence. As a result, the infraspecific classification within sect. *Tuberculata* has remained taxonomically controversial.

The complex species comprises closely related taxa that share morphological, genetic, or ecological similarities and often have ambiguous taxonomic boundaries (He et al. 2022). These complexes typically include subspecies or varieties or may involve hybridization, incomplete lineage sorting, or cryptic diversity—factors that complicate species delimitation using traditional morphology. *Camellia lipingensis*, *C. zengii*, and *C. rhytidocarpa* were first described by Hung-Ta Chang in 1984 (Chang and Ren 1996). The types of *C. lipingensis* and *C. zengii* are confined to Wulong Mountain in Liping county, Guizhou province, China, where they grow in the natural habitats of mixed forests or bamboo groves. *C. lipingensis* is distinguished by narrow lanceolate, thick, leathery leaves, glabrous styles, and 5 cm-diameter flowers. *C. zengii* features villous styles, stamens 1.8–2.4 cm long, 10 segments, and sepals 1–4 cm long. The type specimen of *C. rhytidocarpa*, from Tianping Mountain in Longsheng county, Guangxi province, China, grows on valley areas in the understory of forests. In addition, the straight-line distance between the origins of the three varieties exceeds 200 km. *C. rhytidocarpa* has lanceolate to oblong leaves (8–12 cm long) with 6–7 pairs of unsunken lateral veins. A previous taxonomic study merged these three species into *C. rhytidocarpa* (Min et al., 1993), citing shared traits such as narrowly lanceolate leaves with caudate or acuminate apices, ovate sepals and ovaries, and sparsely pubescent trilobed styles. However, this review relied exclusively on herbarium specimens, with minimal field observation. Moreover, supporting evidence from disciplines such as molecular phylogenetics, palynology, or anatomical morphology is lacking. In recent decades, *Camellia* taxonomy has increasingly incorporated pollen micromorphology, cpDNA, and leaf epidermal features for these species (Jiang et al. 2010; Yan et al. 2024; Ran et al. 2024c).

In recent years, our extensive field investigations at the type localities of these three species revealed consistent disparities in key taxonomic traits of flowers and fruits, indicating that they should not be treated as conspecific. These initial findings prompted a comprehensive, multidisciplinary study integrating detailed morphological (including leaf epidermis and pollen morphology), anatomical, and molecular phylogenetic (cpDNA and ITS) analyses. The congruent evidence from these approaches consistently supports the recognition of three distinct species. Herein, we present a detailed taxonomic revision based on this integrative evidence.

## Materials and methods

### Observation and inspection of type specimens

Type specimen examination was systematically conducted to clarify species boundaries and verify historical taxonomic foundations, utilizing digitized resources from the Herbarium of the Institute of Botany, Chinese Academy of Sciences (PE; http://pe.ibcas.aCamelliacn/peweb/), Sun Yat-sen University Herbarium (SYS; https://eco.sysu.edu.cn/platforms/museum), and the Chinese Virtual Herbarium (CVH; https://www.cvh.ac.cn/index.php). Morphological parameters were rigorously measured using standardized techniques (Ran et al. 2024a), with type specimens of *Camellia lipingensis*, *C. zengii*, and *C. rhytidocarpa* sourced through an original literature review and authoritative digital platforms, including CVH and the National Specimen Resource Sharing Platform (NSII; http://www.nsii.org.cn/2017/home.php) (Wu et al. 2022; Seo et al. 2025). Comprehensive morphological observations and comparative analyses were performed on all the examined samples.

### Material collection

The field materials used for experimental and morphological data measurements in this study were collected through systematic field surveys between 2022 and 2024. Fresh leaves and floral organs collected in the field were preserved via dry ice flash-freezing to ensure sample integrity (Xiao et al. 2024). Voucher specimens were processed following international herbarium standards and deposited in the Herbarium of the Forestry College, Guizhou University (*Camellia lipingensis*: LZ-20231126-7; *Camellia zengii*: LZ-20231127-3; *Camellia rhytidocarpa*: LZ-20240114-4).

### Morphology study

In this study, plant morphological characteristics were systematically investigated through integrated herbarium specimen examinations and field observations, and natural habitats and overall plant morphology were documented before standardized measurements of qualitative and quantitative traits from ≥ 10 voucher specimens per species were conducted. The comprehensive measurements included stem/branch bark coloration and texture, leaf characteristics, floral features, and fruit attributes, with all the parameters measured in triplicate and averaged (Ran et al. 2024b). The standardized dataset (Table S1) was processed using Excel 2025 and subsequently analyzed through principal component analysis (PCA) of 35 morphological variables in R v4.4.2 (R Core Team 2020).

### Leaf epidermis and pollen micromorphology

Fresh leaves and floral buds of *Camellia lipingensis*, *C. zengii*, and *C. rhytidocarpa* were collected for micromorphological analysis. The leaf samples were processed as follows: (1) The samples were fixed in FAA (70% ethanol: acetic acid: formaldehyde = 90:5:5, v/v), rinsed, dissected along the midvein (0.5 cm²), treated with sodium hypochlorite at 30 °C, and sectioned. Epidermal sections were stained with acetate fuchsin and observed under a light microscope to examine the periclinal walls and stomatal apparatus (Jiang et al. 2022; Situngu et al., 2022). (2) The leaf tissue was rinsed with PBS, fixed in EM fixative (2 h, RT), and stored at 4 °C. The tissue blocks were subsequently washed three times in 0.1 M PB (pH 7.4, 15 min each), postfixed in 1% OsO₄ (1–2 h, RT), subsequently washed, dehydrated in an ethanol series, critical-point dried, mounted on aluminum stubs, sputter-coated with gold (30 s), and imaged by SEM (Zhang et al. 2021; Neilands et al. 2023).

Pollen grains (10 per species) from FAA-fixed anthers were acetolyzed, released via microdissection, coated with gold, and examined by SEM (JSM-6490LV). Morphology size (polar/equatorial axes), colpi, exine ornamentation, and wall sculpturing were quantitatively analyzed using ImageTool software (Ocaña et al., 2022; Pan et al. 2022).

### Total DNA extraction, sequencing, assembly, and annotation

Fresh leaves of the three species collected during field surveys were subjected to total DNA extraction using the cetyltrimethylammonium bromide (CTAB) method (Chai et al. 2023; Xing et al. 2023). Following Illumina DNA library preparation standards, paired-end sequencing libraries were constructed with 350-bp insert fragments (Li et al. 2021). DNA quality and concentration were assessed by agarose gel electrophoresis. Qualified fragments were sequenced on the Illumina platform after random fragmentation, end repair, and adapter ligation (Zong et al. 2023).

Raw reads from base-calling (CASAVA) were assembled using SPAdes v.3.15.2 and annotated with CPGAVAS and ORFFinder, with validation via BLASTN/BLASTP alignments. tRNA genes were annotated with ARWEN v1.2 (Zheng et al., 2023; Gu et al. 2024). The chloroplast genome was finalized through online annotation, BLAST alignment, and manual curation using *C. rubituberculata* Chang & Yu (MZ424202) as a reference genome (Xiao et al. 2025). Genome maps were generated with OGDRAW (Yan et al. 2023; Ran et al. 2024c). The sequences were deposited in NCBI GenBank to obtain accession numbers.

### IR boundary expansion and contraction

The chloroplast genome sequences of *Camellia lipingensis*, *C. zengii*, and *C. rhytidocarpa* were screened, and three complete chloroplast genomes were obtained from the samples. The sequences of Theaceae species were downloaded from the NCBI for Biotechnology Information database. IR boundary expansion/contraction analysis was performed, and comparative maps were generated using IRscope (https://irscope.shinyapps.io/irapp/) (Amiryousefi et al. 2018; Xiao et al. 2024).

### Analysis of adaptive nucleotide substitution rates in three species

To assess the selective pressure on the protein-coding genes in the chloroplast genomes of the three species, we calculated their nonsynonymous (Ka) and synonymous (Ks) substitution rates, along with the Ka/Ks ratio, using KaKs_calculator v3.0 (Gutiérrez et al. 2023). The analysis was performed under the MYN model (Zhou et al. 2023). A Ka/Ks ratio > 1 indicates positive selection, 1 suggests neutral evolution, and < 1 reflects purifying selection (Zhang et al. 2006).

### Phylogenetic analysis

The chloroplast genomes of 24 *Camellia* species were retrieved from NCBI, with *Apterosperma oblata* (NC_035641) used as the outgroup (Ran et al. 2024a). Sequence alignment was performed using MAFFT v7, followed by maximum likelihood (ML) tree construction in IQ-TREE v1.6.12. Manual refinement in MEGA X resulted in the selection of optimal substitution models (Smith et al. 2021). MrModeltest v2.3 identified (TVM + F + I) as the best-fit model, and Bayesian inference (BI) trees were reconstructed using MrBayes v3.2.7 (Catalano et al. 2025). Phylogenies based on protein-coding genes and ITS were congruent with those of whole chloroplast genomes. The final trees were visualized with iTOL v5 (Letunicet al. 2021).

## Results

### The morphological characteristics of the three species

**Fig. 1:**
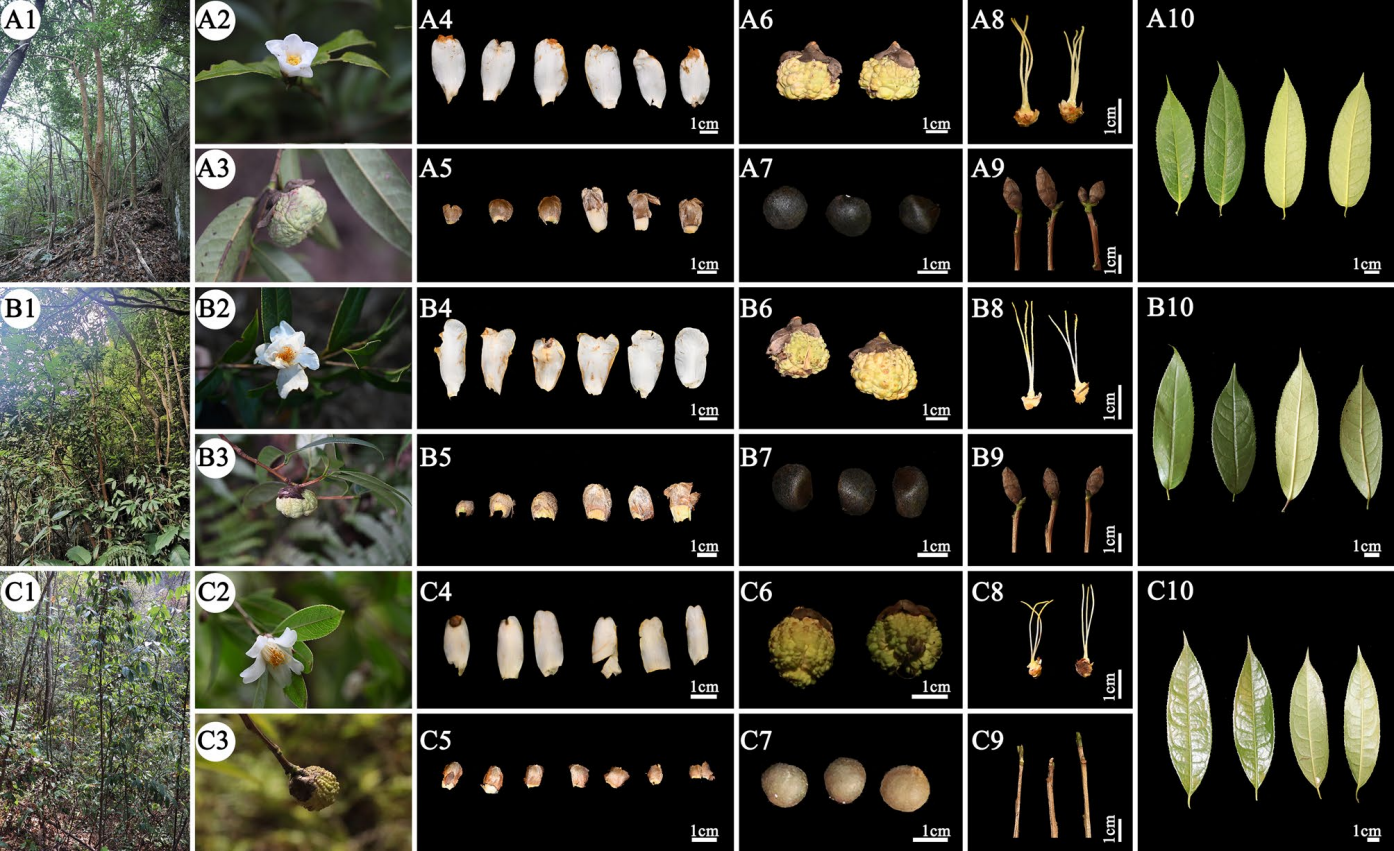
The main morphological characteristics of the three species (**A**: *C. lipingensis*; **B**: *C. zengii*; **C**: *C. rhytidocarpa*. 1: Wild-type habitat, 2: Flower, 3: Fruit, 4: Petals, 5: Calyx, 6: Mature fruit, 7: Seeds, 8: Filaments, 9: One-year branches, 10: Front and back of the leaf)

Systematic morphological comparisons revealed significant phenotypic variations in morphology among the three species (Fig. 1; Table 1). *Camellia lipingensis* and *C. zengii* shared brownish mottled bark exfoliating to green/grey-green surfaces, red-brown quadrangular branchlets, and leathery lanceolate leaves (vs. occasionally oblong in *C. rhytidocarpa*) with acute apices and sparse serrations. The leaf dimensions (6.66–12.48 × 2.16–3.93 cm vs. 5.81–11.60 × 1.17–4.10 cm) and petiole lengths (0.12–1.10 cm vs. 0.30–0.91 cm) overlapped. Floral traits included nearly identical petal dimensions (3.44–5.20 × 1.30–2.00 cm vs. 3.47–4.71 × 1.34–2.21 cm), numbers (6–9 vs. 7–8), ovate sepals, and overlapping ovary/style pubescence. Fruit diameter (2.24–3.15 cm vs. 2.26–3.18 cm) and shape (subglobose) were consistent, with only minor pericarp thickness variation (0.45–0.60 cm vs. 0.43–0.52 cm) falling within intraspecific geographic variation.

*Camellia rhytidocarpa and C. lipingensis* exhibited morphological differences. A comparison of the dimensional measurements of its occasional oblong leaves and acuminate apices revealed distinct differences in leaf dimensions (6.42–12.50 × 2.16–4.21 cm vs. 6.66–12.48 × 2.16–3.93 cm). The relatively dense serration density of *C. rhytidocarpa* leaves may reflect a local adaptation. Floral traits partially overlapped: Petal dimensions (3.20–4.28 × 1.25–1.90 cm vs. 3.44–5.20 × 1.30–2.00 cm), and obtuse sepal apices exhibited intraspecific polymorphism. Fruit diameter (2.24–3.09 cm vs. 2.24–3.15 cm) and shape (subglobose/oblate) were similar, although pericarp thickness was reduced (0.30–0.49 cm), suggesting developmental plasticity. The three species exhibit distinct morphological differences with continuous variation, with *C. rhytidocarpa* being the most abundant, exceeding both *C. lipingensis* and *C. zengii*.

**Table 1:** Comparison of the main morphological characteristics of the three species of sect. *Tuberculata*

Species	*C. lipingensis*	*C. zengii*	*C. rhytidocarpa*
Trunk	Brownish with mottled exfoliation, revealing glaucous green trunks after bark shedding	Mottled brown with inconspicuous and exfoliating rugose bark	Russet bark with conspicuous exfoliation, revealing glaucous green trunks
Branches	Annual branchlets tetragonally ribbed	Annual branchlets tetragonally ribbed	Annual branchlets tetragonally ribbed
Leaf type	Leathery, lanceolate, leaf tail acute	Leathery, lanceolate, leaf tail acute	Leathery, lanceolate or long elliptic, acuminate leaf tail
Leaf length × width (cm^2^)	6.66–12.48 × 2.16–3.93	5.81–11.60 × 1.17–4.10	6.42–12.50 × 2.16–4.45
Petiole length (cm)	0.12–1.10	0.30–0.91	0.43–1.30
Number of petals	6–9	7–8	6–8
Number of calyxes	6–9	7–8	6–8
Sepal	Ovate, apex rounded	Ovate, few rounded, apex rounded	Rounded, apex rounded
Fruit diameter (cm)	2.24–3.15	2.26–3.18	2.24–3.09
Fruit shape	Subglobular	Subglobular	Oblate
Shell thickness (cm)	0.45–0.60	0.43–0.52	0.30–0.49

**Fig. 2:**
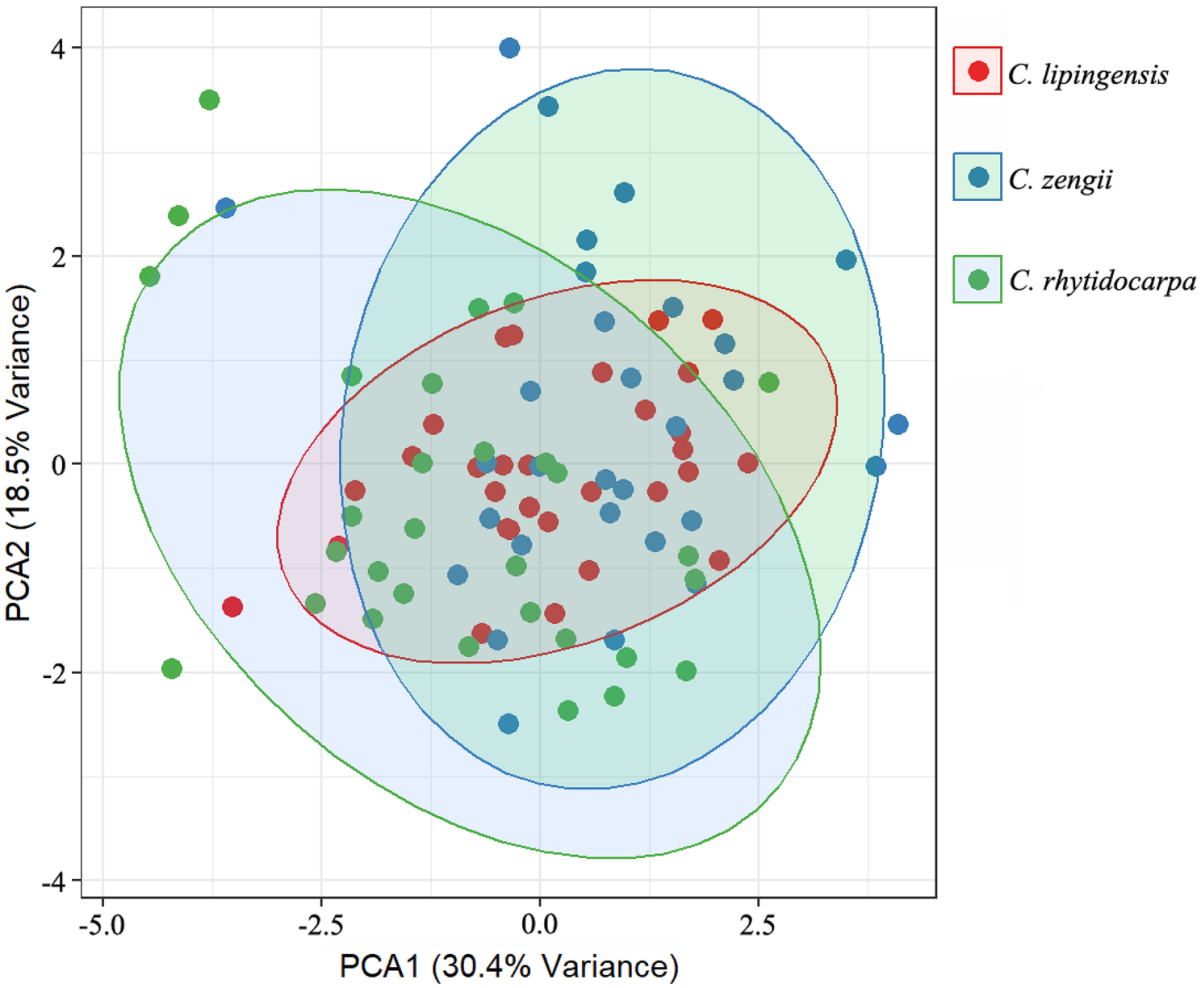
Principal component analysis of the morphological characteristics of the three species. Confidence ellipses indicate the 95% normal distribution range for each group

To assess the taxonomic value of the morphological traits across the three species, we performed a principal component analysis (PCA). The results clearly separated all the samples into three clusters. While *C. lipingensis*, *C. zengii*, and *C. rhytidocarpa* exhibited substantial morphological overlap, with measured traits reflecting a nested relationship among them, *C. rhytidocarpa* displayed the greatest variation (Fig. 2).

### Leaf epidermal micromorphological characteristics

**Fig. 3:**
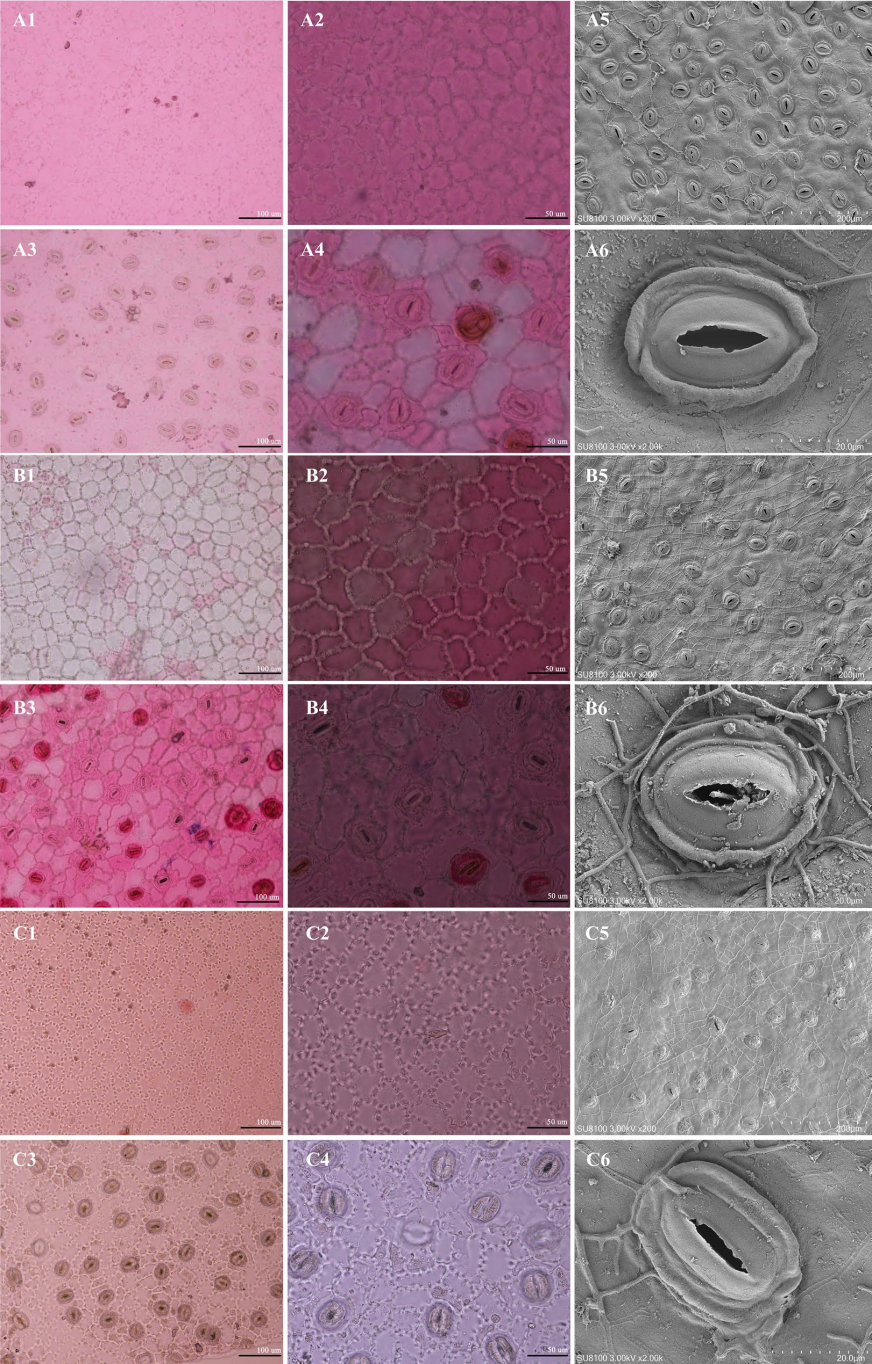
Micromorphological characteristics of the leaf epidermis of three species (**A**: *C. lipingensis*; **B**: *C. zengii*; **C**: *C. rhytidocarpa*. 1, 2: Adaxial surface; 3, 4: Hypodermis; 1, 3: 20×; 2, 4: 40×; 5, 6: stomatal apparatus)

Microscopy revealed (Fig. 3; Table 2) highly conserved yet continuously variable leaf micromorphology across the three species. *Camellia lipingensis*, *C. zengii*, and *C. rhytidocarpa* shared irregular epidermal cells with sinuous anticlinal walls on adaxial surfaces. Crucially, oil glands were absent in *C. lipingensis* and *C. zengii* but were sparse in *C. rhytidocarpa*.

Abaxially, *Camellia lipingensis* and *C. zengii* exhibited sinuous anticlinal walls versus undulate walls in *C. rhytidocarpa*, although all the species presented a tight cell arrangement and anomocytic stomata with reniform guard cells and 3–5 similarly configured subsidiary cells.

**Table 2:** Morphological characteristics of the leaf epidermis of the three species

Species	Shape of stomata	Size of stomata (length/µm × width/µm)	Length / Width	Inner margin of outer rim	Stomatal density (stomata/mm²)	Type of stomatal apparatus
*C. lipingensis*	Long ellipse	40.8–40.9 × 32.5–36.5	1.12–1.25	Shallow waveform	68–86	Oval
*C. zengii*	Long ellipse	39.9–40.8 × 31.9–36.7	1.10–1.26	Shallow waveform	65–85	Oval
*C. rhytidocarpa*	Long ellipse	40.1–41.5 × 31.4–36.7	1.14–1.22	Shallow waveform	62–78	Oval

*Camellia lipingensis* and *C. zengii* shared elliptical stomata with fully overlapping dimensions: Length (40.8–40.9 μm vs. 39.9–40.8 μm), width (32.5–36.5 μm vs. 31.9–36.7 μm), length-to-width ratio (1.12–1.25 vs. 1.10–1.26), and density (68–86 vs. 65–85 stomata /mm²). Both exhibit sinuous inner periclinal walls.

The morphology of *Camellia rhytidocarpa* and *C. lipingensis* showed morphological continuity: The stomatal length (40.1–41.5 μm vs. 40.8–40.9 μm) differs slightly, but width (31.4–36.7 μm vs. 32.5–36.5 μm) and L/W ratio (1.14–1.23 vs. 1.12–1.25). Differences in density were detected (62–78 vs. 68–86 stomata/mm²) (Table S2).

### The microscopic morphological characteristics of the pollen of the three species

**Fig. 4:**
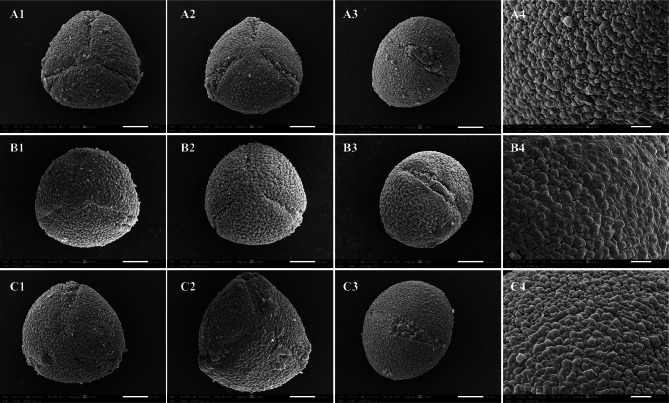
Electron microscopy images of the three species. (**A**: *C. lipingensis*; **B**: *C. zengii*; **C**: *C. rhytidocarpa*)

Palynological evidence (Table 3; Fig. 4) revealed nearly identical pollen characteristics in *Camellia lipingensis* and *C. zengii*. Both species produce subspherical pollen with partially overlapping polar axis (36.7–36.8 μm vs. 36.8 μm) and equatorial axis lengths (40.3 μm vs. 41.3 μm). The P/E ratio (0.90 vs. 0.9) showed complete convergence, whereas the colpus dimensions exhibited minimal divergence: Length (26.3–26.4 μm vs. 26.3 μm), width (7.3 μm vs. 7.4 μm), and a mean L/W ratio of 3.6. Identical granular exine ornamentation and muri-granular complexes further support morphological continuity.

Compared with *C. lipingensis* and *C. zengii*, *Camellia rhytidocarpa* exhibits continuous clinal variation in pollen morphology but maintains substantial overlap. Compared with that of C. zengii (36.8 μm), the oblate-spheroidal pollen of *C. rhytidocarpa* has a slightly longer polar axis (37.2–37.8 μm). In contrast, its equatorial axis (41.3–41.4 μm) and P/E ratio (0.7 vs. 0.9) demonstrate inconsistent variation relative to those of the other taxa. While the granular exine ornamentation is semblable, differences in cell wall thickness (0.5 μm in *C. rhytidocarpa* versus 0.5–0.6 μm in *C. zengii*) further indicate ultrastructural differentiation among these species.

**Table 3:** Pollen morphological characteristics of the three species

Species	Pollen shape	Polar axis (*P*)/µm	Equatorial axis (E)/µm	Polar axis/equatorial axis	Germination groove/µm		
					Length	Width	Length/Width (average data)
*C. lipingensis*	Near-spherical shape	36.7–36.8	40.3	0.9	26.3–26.4	7.3	3.6
*C. zengii*	Near-spherical shape	36.8	41.3	0.9	26.3	7.4	3.6
*C. rhytidocarpa*	Near-spherical shape	37.2–37.8	41.3–41.4	0.7	27.7–27.7	7.4	3.3

### Characteristics of the chloroplast genome

**Fig. 5:**
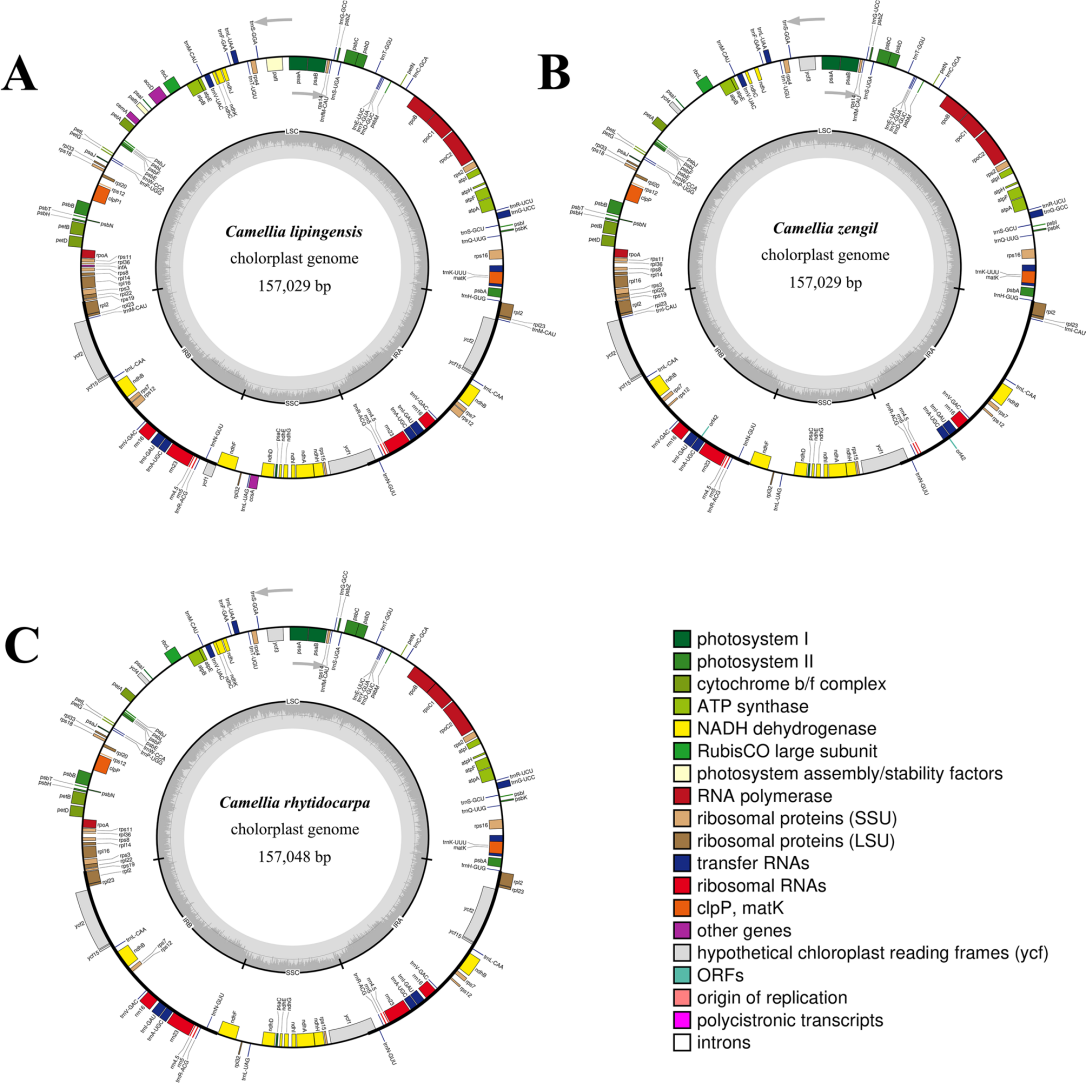
Chloroplast genome maps of the three species. (**A**: *C. lipingensis*; **B**: *C. zengii*; **C**: *C. rhytidocarpa*)

Integrated molecular analyses (Table 4; Fig. 5) revealed high chloroplast genome conservation among *Camellia lipingensis*, *C. zengii*, and *C. rhytidocarpa*. The genome sizes were 157,029 bp (*C. lipingensis*/*C. zengii*) and 157,048 bp (*C. rhytidocarpa*), with identical GC contents (37.3%). LSC/SSC/IR lengths were conserved in *C. lipingensis* and *C. zengii* (LSC 86,630 bp, SSC 18,281 bp, IR 52,118 bp), whereas *C. rhytidocarpa* showed minimal LSC expansion (86,648 bp) and stable SSC (18,282 bp).

The regional GC content exhibited negligible variation: The LSC ranged from 35.31 to 35.32%, the SSC ranged from 30.61%, and the IR ranged from 42.94 to 42.97%. The gene inventories were identical: 132 total genes (87 protein-coding, 37 tRNA, and 8 rRNA). Slight divergence occurred only in the third-codon position GC content (29.40-29.47%). IR boundary analysis confirmed conserved gene positioning (*rpl22*, *rpl2*, *psbA*). The expansion of the LSC in *C. rhytidocarpa* may represent potential ecological adaptation.

**Table 4:** Genome-wide characteristics of chloroplasts in three species

Species	*C. lipingensis*	*C. zengii*	*C. rhytidocarpa*
Genome size (bp)	157,029	157,029	157,048
GC (%)	37.3	37.3	37.3
LSC size (bp)	86,630	86,630	86,648
SSC size (bp)	18,281	18,281	18,282
IR size (bp)	52,118	52,118	52,118
GC in LSC (%)	35.32	35.32	35.31
GC in SSC (%)	30.61	30.61	30.61
GC in IR (%)	42.94	42.94	42.97
GC in CDS (%)	37.61	37.61	37.61
1st position GC (%)	45.21	45.21	45.37
2nd position GC (%)	37.9	37.9	38.04
3rd position GC (%)	29.47	29.45	29.40
Length of CDS	79,100	79,100	79,100
Number of genes	132	132	132
Number of CDS	87	87	87
Number of tRNA	37	37	37
Number of rRNA	8	8	8

**Fig. 6:**
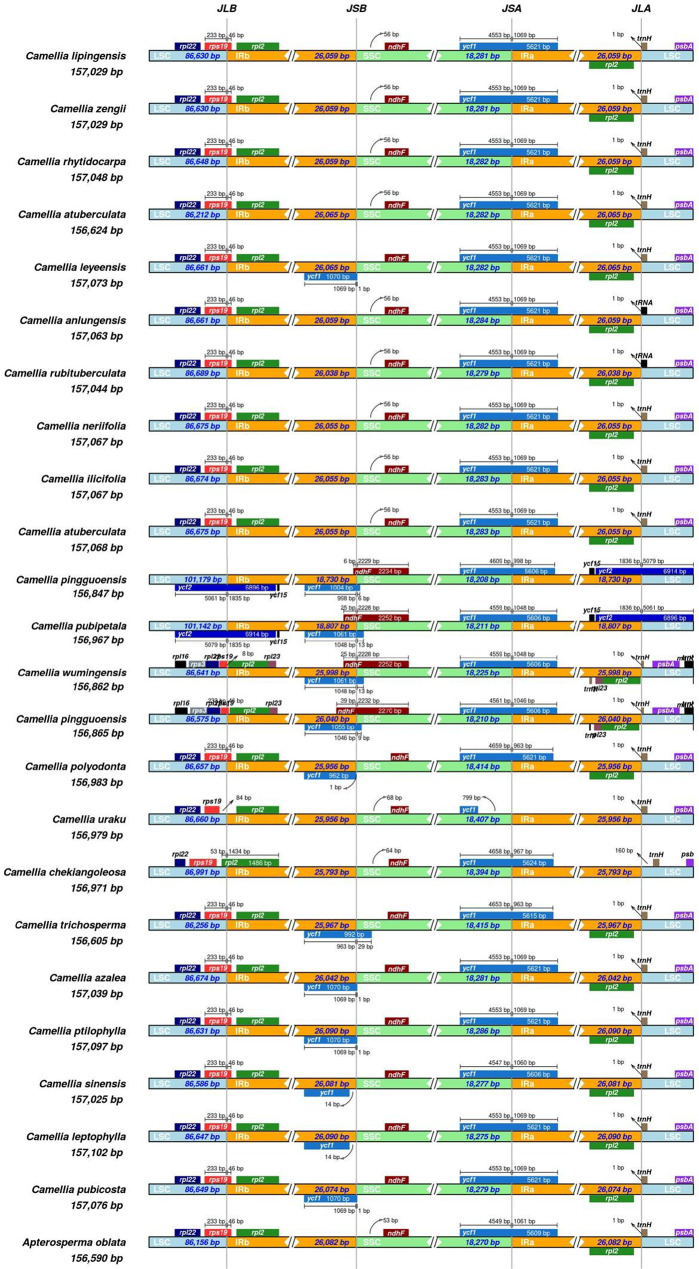
Analysis of the contraction and expansion of IR boundaries in twenty-four species

IR boundary contraction/expansion analysis (Fig. 6) confirmed the conserved positioning and lengths of the *rpl22*, *rpl2*, and *psbA* genes in *Camellia lipingensis*, *C. zengii*, and *C. rhytidocarpa*. Minimal interspecific divergence occurred, with slight variations in the length of the LSC. Comparative assessment with *C. rubituberculata* and *C. anlungensis* revealed distinct IR boundary initiation patterns: The focal species initiated at the *trnH* gene, whereas *C. rubituberculata* and *C. anlungensis* initiated at tRNA genes. All twenty-four species exhibited similar contraction/expansion trends. Despite the conserved gene architecture, subtle interspecific differences indicate lineage-specific adaptations. These structural variations likely reflect ecological selection pressures, influencing genomic organization and functional expression.

### Selection of stress analysis results

A total of 93 protein-coding genes from the chloroplast genomes of *Camellia lipingensis*, *C. zengii*, and *C. rhytidocarpa* were analyzed to estimate nucleotide substitution rates. Using the MYN model in KaKs_calculator v3.0, we calculated non-synonymous (Ka) and synonymous (Ks) substitution rates and their ratio (Ka/Ks). Among the genes compared, eight yielded calculable results: *accD*, *atpA*, *ndhC*, *ndhD*, *psaI*, *psaJ*, *psbA*, and *psbB*. Two of these genes (*psbA* and *psaJ*) presented a Ka/Ks > 1, indicating positive selection (Table 5). These findings suggest limited evidence for ecological adaptation in these species in response to environmental pressures.

**Table 5:** Ka/Ks values for protein-coding gene alignments among the three species

Species 1	Species 2	gene	Ka	Ks	Ka/Ks
*C. rhytidocarpa*	*C. zengii*	*accD*	0	0.003	0
*C. lipingensis*	*C. rhytidocarpa*	*atpA*	0	0.003	0
*C. rhytidocarpa*	*C. zengii*	*atpA*	0	0.003	0
*C. rhytidocarpa*	*C. zengii*	*atpA*	0	0.003	0
*C. rhytidocarpa*	*C. zengii*	*ndhC*	0	0.002	0
*C. lipingensis*	*C. rhytidocarpa*	*ndhD*	0	0.002	0
*C. lipingensis*	*C. rhytidocarpa*	*psaI*	0.001	0.002	0.743
*C. lipingensis*	*C. zengii*	*psaI*	0.001	0.002	0.744
*C. rhytidocarpa*	*C. zengii*	*psaJ*	0.020	0.020	1.034
*C. lipingensis*	*C. rhytidocarpa*	*psbA*	0.020	0.020	1.034
*C. lipingensis*	*C. zengii*	*psbA*	0.023	0.023	0.988
*C. lipingensis*	*C. zengii*	*psbA*	0.020	0.020	1.034
*C. lipingensis*	*C. rhytidocarpa*	*psbB*	0	0.019	0
*C. lipingensis*	*C. zengii*	*psbB*	0	0.019	0

### Phylogenetic analysis

**Fig. 7:**
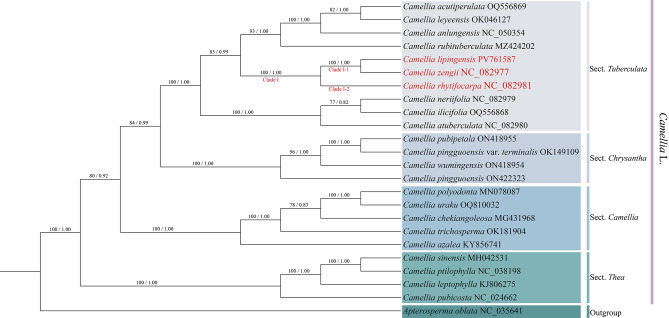
Construction of a phylogenetic tree based on the chloroplast genome (maximum likelihood (ML) and Bayesian (BI) trees; BS ≥ 50% and PP ≥ 0.95 are indicated above the branches as BS/PP)

**Fig. 8:**
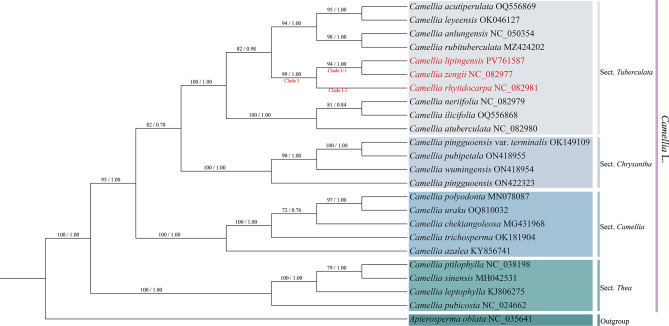
Construction of a phylogenetic tree based on the PCGs (maximum likelihood (ML) and Bayesian (BI) trees; BS ≥ 50% and PP ≥ 0.95 are indicated above the branches as BS/PP)

**Fig. 9:**
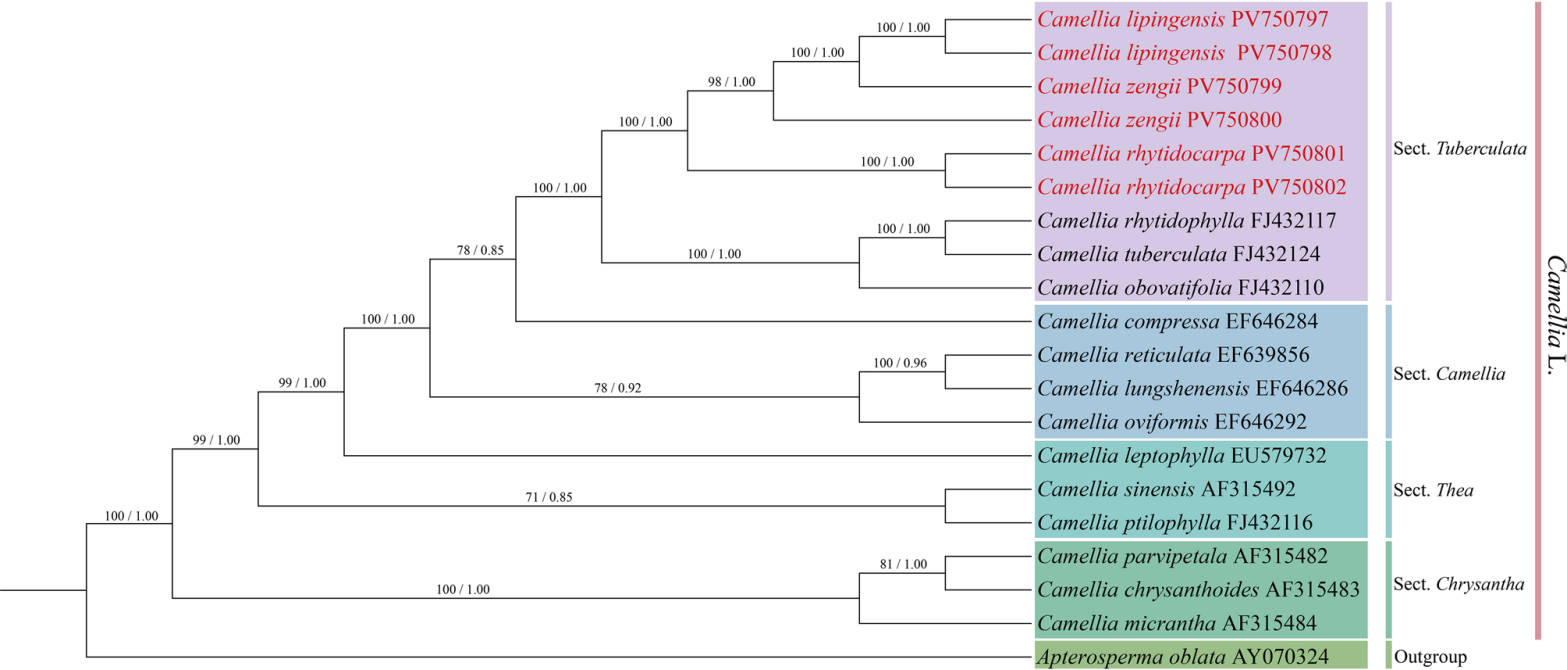
Construction of a phylogenetic tree based on the ITS (maximum likelihood (ML) and Bayesian (BI) trees; BS ≥ 50% and PP ≥ 0.95 are indicated above the branches as BS/PP)

Phylogenomic analysis (Fig. 7) revealed that the support rates of *Camellia lipingensis*, *C. zengii*, and *C. rhytidocarpa* were high for Clade I (ML = 100; BI = 1.00). Specifically, *C. lipingensis* and *C. zengii* formed a monophyletic clade in clade I-1 (ML = 100, BI = 1.00), whereas *C. rhytidocarpa* occupied clade I-2. Congruently, two phylogenetic trees based on protein-coding genes (Fig. 8) and ITS sequences (Fig. 9) consistently resolved all three species within sect. *Tuberculata*, clustering them on a shared branch with high statistical support.

### Taxonomic treatment

**Fig. 10:**
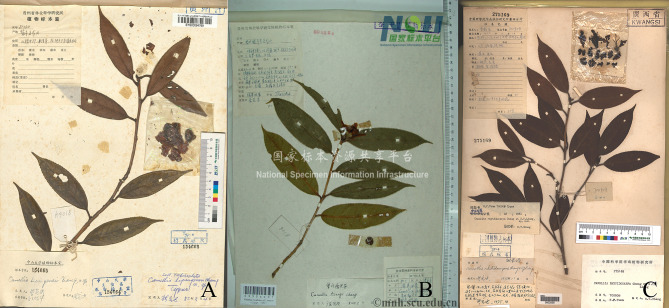
Type specimen information of the three species. (**A**: *C. lipingensis*; **B**: *C. zengii*; **C**: *C. rhytidocarpa.* Figure **B** was obtained from the National Specimen Platform (http://www.nsii.org.cn/2017/home.php) and was found to be a type of specimen after verification

The comprehensive evidence from morphology, anatomy, palynology, and molecular phylogenetics all support the synonymization of *Camellia zengii* under *C. lipingensis*, with the latter name being retained in accordance with the principle of priority in botanical nomenclature. In contrast, *C. rhytidocarpa* should be considered a distinct species. (Fig. 10).

*Camellia lipingensis* Hung T. Chang, Acta Sci. Nat. Univ. Sunyatseni 23(2): 78. 1984.

**Type**: CHINA. Guizhou Province: Liping County, Wulong Mountain, s. *Camellia* 808, 81,064 (holotype SYS; isotype GZFI).

= *Camellia zengii* Hung T. Chang, Acta Sci. Nat. Univ. Sunyatseni 23(2): 77. 1984. syn. nov.

**Type**: CHINA. Guizhou Province: Liping County, Wulong Mountain, s. *Camellia* 8017, 8018 (holotype GZFI; isotypes SYS).

#### Botanical description

Small macrophanerophytes with glabrous, lustrous young branches. Leaves thickly coriaceous, lanceolate, 6–12 cm long × 2–4 cm wide; apex caudate-acuminate, base broadly cuneate to rounded; adaxially greenish and slightly lustrous when dry, abaxially fulvous and glabrous except for sparse villous hairs along midvein; lateral veins 5–10 pairs prominently raised on both surfaces, reticulation obscure; margin sharply serrate; petiole 5–11 mm. Flowers white, terminal, subsessile, to 5 cm diameter. Outer bracts 4, scarious, apex apiculate and pubescent. Inner bracts ovate, 1–1.4 cm long, apex acute, margin scarious and densely pubescent. Petals 10, oblong to ovate, 3.5 cm long. Stamens 3 cm long, outer filaments nearly free. Ovary 3-loculed, densely pubescent; styles 3, 3.5 cm long, pubescent. Capsule oblate, tuberculate, 2.5–3 cm diameter, 3-loculed; seeds 1 per locule, densely tomentose. Flowering from August to September.

#### Distribution and habitat

**Fig. 11:**
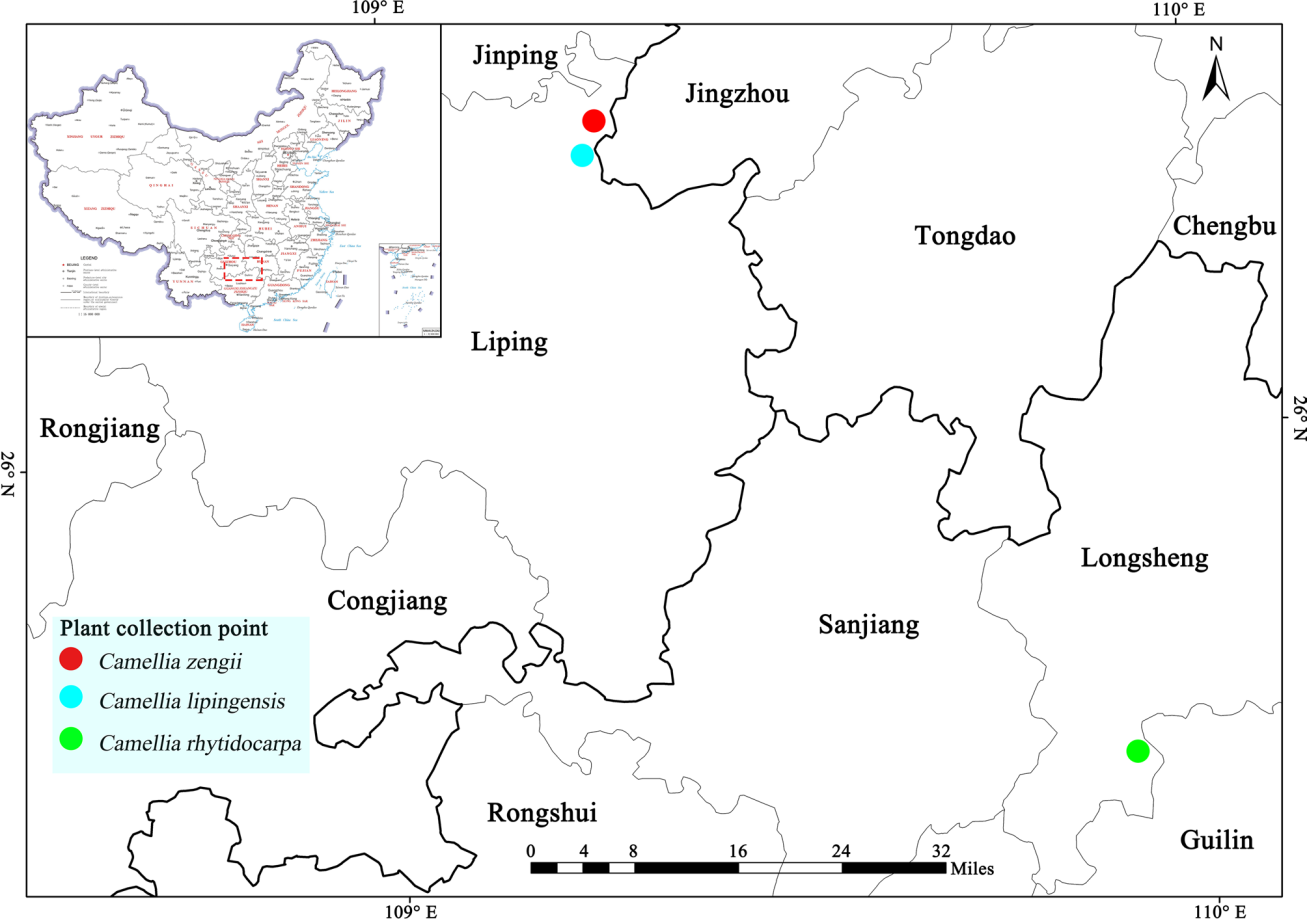
Map of collection loci of *C. lipingensis*, *C. zengii* and *C. rhytidocarpa*

*C. lipingensis* and *C. zengii* are endemic to Liping county, Guizhou province. Their typical habitats include Wulong Mountain (Qiutuan village), Cenba village, and Huangtianbang, where they thrive in mountain ecosystems at 1000–1200 m elevation, predominantly on slope gradients of 15–45 °C (Fig. 11).

*Camellia rhytidocarpa* Hung T. Chang & S. Y. Liang in Chang, Tax. Gen. *Camellia*: 49. 1981. syn. nov.

**Type**: CHINA. Guangxi Zhuang Autonomous Region: Longsheng County, Tianpingshan Village, s. *Camellia* 700, 908 (holotype SCBI; isotype PE).

#### Botanical description

A perennial shrub or small tree with glabrous young branches. Leaves thickly coriaceous, oblong or lanceolate, 6–12 cm long and 2.5–4.5 cm wide; apex acuminate, base obtuse to subrounded; adaxial surface olive-green when dried, glossy, abaxial surface yellowish-green, glabrous; lateral veins 6–7 pairs, slightly impressed adaxially and prominent abaxially; margin sharply serrulate, with teeth spaced 2–3 mm apart; petiole 8–12 mm, glabrous. Flowers terminal, white, sessile; bracts and sepals in 10 segments, gradually increasing in size, the outermost 3 semilunar, the inner 7 ovate, 1–1.4 cm long, sericeous abaxially; petals 6–8, 3.2–4.3 cm long, basally connate for approximately 2/5 of their length, free portions obovate, the outermost 1 or 2 petals slightly shorter and pubescent abaxially; stamens 2–2.2 cm long, outer whorl with filaments fused up to 1.3 cm at base, both free parts and filament tube glabrous, filament tube adnate to petals at base; ovary pubescent; styles 3–4, 2 cm long, glabrous. Capsule globose, tuberculate, 2–2.5 cm in diameter, 1-loculed with 1 seed, or occasionally bilobed and 2-loculed; seeds spherical, pubescent. Flowering occurs from November to December.

#### Distribution and habitat

*Camellia rhytidocarpa* is restricted to Tianping Mountain village, Longsheng County, Guangxi Zhuang Autonomous Region. This species occupies gentle valleys, forest understories, and riparian zones within the same altitudinal range (1000–1200 m) (Fig. 11).

## Discussion

The taxonomic controversy surrounding *Camellia lipingensis*, *C. zengii*, and *C. rhytidocarpa* has long centered on intraspecific variation in floral and leaf morphology (Min et al., 1993; Chang 1996). In this study, morphological, anatomical, palynological, and molecular phylogenetic evidence is integrated with long-term field surveys and type specimen examinations to clarify their delimitation. These findings indicate that morphological traits such as leaf shape, petiole length, and fruit size are consistent with their classification as two distinct species. Such trait stability represents a phenotypic response to heterogeneous habitats in terms of plant taxonomy (Williams 2010). The consistent bark, branchlets, leaves, flowers, and fruits strongly support the merging of *C. lipingensis* and *C. zengii*. In contrast, *C. rhytidocarpa* remains distinct because of its leaf serration, shape, and thinner pericarp.

In terms of leaf epidermal micromorphology, *Camellia rhytidocarpa* has a sparse distribution of oil glands. Both *C. lipingensis* and *C. zengii* exhibit sinuous anticlinal walls, whereas *C. rhytidocarpa* displays undulate walls. The overlapping stomatal dimensions and shared sinuous wall structure strongly support the merging of *C. lipingensis* and *C. zengii*. In contrast, the morphological continuity and gland distribution patterns of *C. rhytidocarpa* substantiate its status as a distinct species. These micromorphological distinctions align with the recognition of *C. rhytidocarpa* as separate from the *C. lipingensis*-*C. zengii* complex. This conclusion is consistent with the principle that intraspecific variation manifests gradually, whereas interspecific differences remain discernible (Pinedo et al. 2016; Longhi et al. 2024). Additionally, stomatal micromorphology data corroborate Barrio’s (2023) finding that leaf morphological conservatism is significantly correlated with local environmental selection pressure.

Type specimen verification and wild population sampling are essential for species delimitation (Xue et al. 2018; Xu et al. 2023). In this study, when the digital specimen banks (such as CVH, GBIF, and *Camellia*) and the type of specimens in the collection were systematically verified, some information deficiencies were detected in the existing samples, such as the absence of complete morphological characteristics of flowers and fruits. Such deficiencies have historically led to overreliance on fragmented characteristics, neglecting population-level trait continuity (Bebber et al. 2010). By integrating voucher specimens with field-collected data, we reconstructed morphological profiles and taxonomic status for these taxa within sect. *Tuberculata*, as corroborated by Bossa-Castro et al.’s (2024) finding that > 30% of global herbarium specimens lack critical diagnostic traits, necessitating field validation.

The diversity of pollen morphology is the result of plants adapting to different environmental and ecological conditions over the course of long-term evolution (Mander et al. 2021). The continuous variation in the pollen polarization ratio and outer wall ornamentation (coarse net-ridge-papilla composite structure) of *Camellia lipingensis*, *C∙ zengii*, and *C. rhytidocarpa* is consistent with intraspecific pollen polymorphism (Gamal 2025), whereas the flattened spherical pollen and significantly reduced papilla density of *C. rhytidocarpa* point to an independent evolutionary path. This difference may reflect their ability to adapt to different pollination environments, with their distribution in drier, hotter regions potentially driving the restructuring of pollen outer wall structures. Additionally, the regular differences in the distribution of germination pores further reinforce their taxonomic independence (Amstutz et al. 2024).

Molecular phylogenetic evidence provides critical support for taxonomic revision (Wei et al. 2023; Abe et al. 2024; Qin et al. 2024). Chloroplast genome analysis revealed high sequence similarity between *Camellia lipingensis* and *C. zengii*, whereas notable differences distinguished them from *C. rhytidocarpa*. Phylogenetic reconstruction further revealed that *C. lipingensis* and *C. zengii* formed a well-supported clade (ML = 100; BI = 1.00), whereas wrinkled-fruit tea (*C. rhytidocarpa*) constituted a distinct lineage. These results support the recognition of *C. lipingensis* and *C. zengii* as conspecifics, with *C. rhytidocarpa* representing a separate species. Nevertheless, it is inferred that *C. rhytidocarpa* shares a closer phylogenetic affinity with both *C. lipingensis* and *C. zengii* in terms of early evolutionary history. These findings align with the view proposed by Chang et al. (2021) that chloroplast genomes remain largely conserved among closely related species. Despite the sympatric distribution of *C. lipingensis* and *C. zengii*, the nuclear ITS phylogeny confirms their genetic homogeneity, validating the reliability of multigene analysis in terms of taxonomy reported by Wang et al. (2025).

Geographic isolation and niche differentiation drive interspecific divergence (Lindelof et al. 2020). *Camellia lipingensis* and *C. zengii* cooccur in similar mountainous habitats (1000–1200 m) in Liping county, Guizhou, whereas *C. rhytidocarpa* is restricted to Longsheng county, Guangxi. The differences in leaf distribution points indirectly demonstrate the characteristic species differentiation within distinct environmental zones. Habitat heterogeneity likely drives morphological adaptations through natural selection. The PCA results indicate that while *C. rhytidocarpa* has slightly larger leaves and longer petioles (0.43–1.30 cm), its smaller corolla size significantly distinguishes it from both *C. lipingensis* and *C. zengii*. However, the floral traits, such as petal number and the presence of complete bisexual flowers, did not markedly differ between these two varieties. Environmental variation elicits differential phenotypic responses within species; e.g., acuminate leaf apices may reduce transpiration in drier microhabitats (Liu et al. 2025).

Molecular phylogenetic analyses revealed that the chloroplast genomes of *Camellia lipingensis* and *C. zengii* measure 157,029 bp, distinguishing them from that of *C. rhytidocarpa* (157,048 bp). *C. lipingensis* and *C. zengii* share identical LSC, SSC, and IR region lengths (86,630 bp, 18,281 bp, and 52,118 bp, respectively), whereas *C. rhytidocarpa* has a slightly expanded LSC region (86,648 bp). These findings align with earlier observations by Mondini et al. (2009), who reported that chloroplast genomic divergence among closely related species is generally subtle. In the phylogenetic tree, *C. lipingensis* and *C. zengii* formed a single clade, whereas *C. rhytidocarpa* formed a distinct subclade. This finding is consistent with the asynchrony of morphological-genetic differentiation demonstrated by Zou et al. (2021) in Rhododendron L., which suggests that phenotypic plasticity may play a dominant role in the formation of morphological differences.

## Conclusion

This study pioneers an integrated multidisciplinary analysis of *Camellia lipingensis*, *C. zengii*, and *C. rhytidocarpa*, systematically resolving their morphoanatomical traits, palynological patterns, and molecular phylogenetics. This research revealed the synonymization of *C. lipingensis* and *C. zengii* while affirming *C. rhytidocarpa* as a distinct taxon. This revision resolves the long-standing synonymy question of the three species in sect. *Tuberculata.* The revealed morpho-molecular coevolution further provides a theoretical foundation for assessing intraspecific variation, genetic diversity patterns, and adaptive evolution in *Camellia* L.

## Supplementary Information

Below is the link to the electronic supplementary material.

Supplementary Material 1

Supplementary Material 2

## Data Availability

GenBank accession numbers: PV761587, PV750797, PV750798, PV750799, PV750800, PV750801, PV750802. The Appendix Table for this article can be found online.

## Abbreviations

FAA: Formalin–Acetic Acid–Alcohol
PBS: Phosphate-Buffered Saline
SEM: Scanning electron microscopy
CTAB: Cetyltrimethylammonium bromide
cpDNA: Chloroplast DNA
LSC: Large Single-Copy region
SSC: Small Single-Copy region
IR: Inverted Repeat
GC: Guanine-Cytosine content
CDS: Coding DNA Sequence
tRNA: Transfer RNA
rRNA: Ribosomal RNA
ML: Maximum Likelihood
BI: Bayesian inference
PP: Posterior Probability
BS: Bootstrap Support
PCGs: Protein-Coding Genes
ITS: Internal transcribed spacer
PCA: Principal component analysis
NCBI: National Center for Biotechnology Information
GBIF: Global Biodiversity Information Facility
